# Comparative Kinetic Study and Microwaves Non-Thermal Effects on the Formation of Poly(amic acid) 4,4′-(Hexafluoroisopropylidene)diphthalic Anhydride (6FDA) and 4,4′-(Hexafluoroisopropylidene)bis(*p*-phenyleneoxy)dianiline (BAPHF). Reaction Activated by Microwave, Ultrasound and Conventional Heating

**DOI:** 10.3390/ijms12106703

**Published:** 2011-10-11

**Authors:** Hugo Mendoza Tellez, Joaquín Palacios Alquisira, Carlos Rius Alonso, José Guadalupe López Cortés, Cecilio Alvarez Toledano

**Affiliations:** 1 Laboratorio de Fisicoquıímica Macromolecular, Facultad de Química, Universidad Nacional Autónoma de México, Edificio “D” 108, Ciudad Universitaria, México 04510, D.F., C.P., Mexico; E-Mail: polylab1@servidor.unam.mx; 2 Laboratorio 204 de Química Orgánica, Facultad de Química, Universidad Nacional Autónoma de México, Ciudad Universitaria, México 04510, D.F., C.P., Mexico; E-Mail: riusal@hotmail.com; 3 Instituto de Química, Universidad Nacional Autónoma de México, Circuito Exterior, Ciudad Universitaria, México 04510, D.F., Mexico; E-Mails: jglcvdw@servidor.unam.mx (J.G.L.C.); cecilio@servidor.unam.mx (C.A.T.)

**Keywords:** kinetic, non thermal microwave effects, activation energy, poly(amic acid)

## Abstract

Green chemistry is the design of chemical processes that reduce or eliminate negative environmental impacts. The use and production of chemicals involve the reduction of waste products, non-toxic components, and improved efficiency. Green chemistry applies innovative scientific solutions in the use of new reagents, catalysts and non-classical modes of activation such as ultrasounds or microwaves. Kinetic behavior and non-thermal effect of poly(amic acid) synthesized from (6FDA) dianhydride and (BAPHF) diamine in a low microwave absorbing *p*-dioxane solvent at low temperature of 30, 50, 70 °C were studied, under conventional heating (CH), microwave (MW) and ultrasound irradiation (US). Results show that the polycondensation rate decreases (MW > US > CH) and that the increased rates observed with US and MW are due to decreased activation energies of the Arrhenius equation. Rate constant for a chemical process activated by conventional heating declines proportionally as the induction time increases, however, this behavior is not observed under microwave and ultrasound activation. We can say that in addition to the thermal microwave effect, a non-thermal microwave effect is present in the system.

## 1. Introduction

Polyimides (PI)s possess the cyclic imide and aromatic groups in the main chain and they are some of the best engineering plastics, because of their high thermal and oxidative stability, their good mechanical properties and excellent chemical resistance to dilute acids and organic solvents. Polyimides have been used in nanofiltration [1], hydrogen purification [2], fuel cells [3], adhesive [4], and solar cells [5].

PIs are usually prepared by the so-called two-step method in which a dianhydride and a diamine are allowed to undergo condensation polymerization to form poly(amic acid) precursor, and subsequently the precursor is converted thermally or chemically into the final polyimide, but the reaction usually takes a long time, 20 hours or more. To find effective green chemistry alternative methods for synthesis of PIs, new techniques can be explored with a view to decreasing the reaction time and reaction temperature. Among these new techniques, the implementation of microwaves (MW) and ultrasound (US) energy are an emerging field of research. Ultrasound (US) has been applied in synthesis of inorganic-organic nanocomposite of polyacrylamide (PAM) and gamma-zirconium phosphate (γ-ZrP) [6], isoxazoles [7], polymeric materials as polyamide-6 (Nylon 6) [8,9] and polymethacrylate [10]. Microwave energy has being investigated as a method for processing resins, such as polyacrylamide [11,12] polyamides [13–15] polymethylmethacrylate [16] and more recently polyimides, because of its shorter processing time, its improved energy utilization, and the potential for lower processing temperatures and improved product uniformity. Thus the use of microwave energy for the assembly of polyimides has had great acceptance, for the synthesis of poly(piromellitidimides) by means of monomers of nylon-salt-type [17,18], polyimides with nonlinear optical properties [19–22], poly (ester-imide) [23], and poly (amide-imide) [24–27].

Microwave irradiation tends to produce reaction accelerations as a consequence of the specific heating rate which can not be reproduced by classical heating.

Recently, the synthesis and characterization of two aromatic polyimides obtained from pyromellitic dianhydride (PMDA) and 4,′4 -(hexafluoroisopropylidene)diphthalic anhydride (6FDA) monomers under microwave irradiation was reported. The microwave activation reduce the polymerization times and leads to an acceleration in the chemical process in comparison with the same reaction activated by conventional heating [28].

The observed reaction rate enhancements are explained primarily as a result of a purely thermal/kinetic effect, *i.e*., a “thermal microwave effect”. However, some authors suggest the possibility of a “non-thermal microwave effect” [29,30]. These effects can be rationalized by consideration under the Arrhenius Equation and are reflected in modifications on each of the terms in this model. Thus, the pre-exponential factor A, which depends on the vibration frequencies of the molecules, can be affected by the microwave field, increasing the probability of effective molecular collisions. It could be postulated that activation energy,Δ *G*_a_, can be possibly reduced through a change of the entropy of the system [31].

The aim of this work is to investigate the kinetic behavior of poly(amic acid) formation, synthesized from 4,4′-(hexafluoroisopropylidene) diphthalic anhydride (6FDA) and 4,4′-(hexafluoroisopropylidene) bis(*p*-phenyleneoxy) dianiline (BAPHF) in a low microwave absorbing solvent as *p*-dioxane at low temperature range of 30, 50, 70 °C. According to our previous experimental evidence *p*-dioxane is a low microwave absorbing solvent. A series of experiments were performed by Lidström *et al*. [32] in which two samples containing water and *p*-dioxane, respectively, were heated in a single-mode microwave cavity at a fixed radiation power of 150 watts. Experiments were carried out at an initial temperature of 25 °C. Temperature of water and *p*-dioxane was recorded for different irradiation times and with this information, a comparative plot of temperature *versus* irradiation time was prepared. This plot shows that water sample is more sensitive to microwave irradiation than *p*-dioxane solvent. For a fixed dielectric heating time of 30 seconds the final temperature in the water sample was 132 °C while for the same irradiation time the final temperature in the *p*-dioxane sample was 27 °C. So, it is clear that *p*-dioxane lacks the dipole characteristics, dipolar moment *μ* = 0.45 D, necessary for microwave dielectric heating, while water, which has a large dipole moment (1.84 D), heats readily under microwave irradiation. This is the reason why *p*-dioxane solvent is considered a low microwave absorbing medium for chemical reactions [29,33], since it has a low dipolar moment compared to the classical high microwave absorbing dimethylformamide (DMF) (*μ* = 3.86 D), DMAc (*μ* = 3.7 D) solvents used in this reaction.

The combination of a low temperature and solvent allowed us to look for experimental evidence of non-thermal effects, due to microwave activation of the reaction. Experimental design was made to provide evidence about deviations on kinetic parameters behavior related to Arrhenius equation: reaction temperature, *T* [°C], kinetic rate constant behavior at three temperatures, *k* [L mol^−1^ s^−1^], activation energy of the reaction, Δ *G* [kJ mol^−1^], pre-exponential factor, A [L mol^−1^ s^−1^] and induction time behavior on the polycondensation reaction, *t*_ind_ [min].

The kinetic parameters were studied with a good precision in temperature and microwave power provided by the scientific microwave unit.

This is a comparative study, where rate constants, activation energies, pre-exponential factors and induction time values obtained by microwave irradiation, ultrasound irradiation and conventional heating techniques are compared for the purpose of observation to find evidence of non-thermal effects on the reaction system.

There is little information in the literature regarding non-thermal effects on the kinetics of the reactions activated by microwaves. So this study can help to clarify the benefits of the application of microwaves to produce polymers in short reaction times and good selectivity. The use of ultrasound and microwave energy in the preparation of poly(amic acid) by a condensation reaction, shows several advantages related to the eco-friendly approach, termed green chemistry.

## 2. Experimental

### 2.1. Materials

High purity 4,4′-(hexafluoroisopropylidene)diphthalic anhydride (6FDA) 99% and ′4,4 - (hexafluoroisopropylidene)bis(*p*-phenyleneoxy)dianiline (BAPHF) 97%, 1,4-dioxane ≥99 % were purchased from Sigma-Aldrich.

Commercial *p*-dioxane was dried with zeolites A4 for 12 h before use.

Reactions were carried out in a Discovery Explorer Hybrid monomodal microwave oven with 300 watts of maximum power, temperature control, and Ultrasonic Processor GEX a frequency of 20,000 Hz and 130 watts of maximum power.

### 2.2. General Procedure of Polymerization Reaction

Polymerization reactions were carried out as follows. An equimolar mixture of the dianhydride/diamina (1 mmol/1 mmol) and the appropriate amount of solvent was poured in a 35-mL glass reactor tube. The homogeneous mixture with constant stirring was activated by conventional heating (CH), microwave irradiation (MW) or ultrasound (US) for a set time, at the following temperature settings 30, 50, 70 °C. The temperature was recorded by a thermocouple and controlled to ±2 °C. The thermocouple was set into the polymerization system for reaction activated by CH and US. When the reaction mixture was irradiated with microwaves, temperatures were recorded and controlled automatically with an infrared probe. The thermometer is part of the microwave oven and shows the actual temperature of the reaction mixture. All operations were done under atmospheric pressure. The resulting polymer solution was poured into 10 mL of distilled water. The solid polymer substance was separated from the liquid, washed with water and methanol, and dried at 30 °C in an electrical oven for 4 h. Poly(amic acid) PAA was obtained as a white solid.

All polymer samples were identified with one letter and two digits, according to the next description: first letter corresponds to the activation method used, in this case conventional heating (CH), microwave irradiation (MW) or ultrasound irradiation (US), the first number corresponds to the temperature selected for reaction activation and a second number corresponds to the polymerization time, in minutes. For example, sample C707, stands for poly(amic acid) (6FDA-BAPHF) synthesized by conventional heating in *p*-dioxane, at 70 °C and 300 min reaction time.

### 2.3. Methods of Polymer Characterization

1. Fourier transform infrared spectra (FTIR) of our polymer samples were recorded in a FTIR-Spectrum 400 Perkin-Elmer instrument.
2. The solubility of poly(amic acid) samples was studied in nine different solvents dissolving approximately 10 mg polymer sample in three drops of each solvent at constant temperature of 25 °C.

## 3. Results and Discussion

### 3.1. FTIR Poly (Amic Acid) Characterization

**Figure 1:**
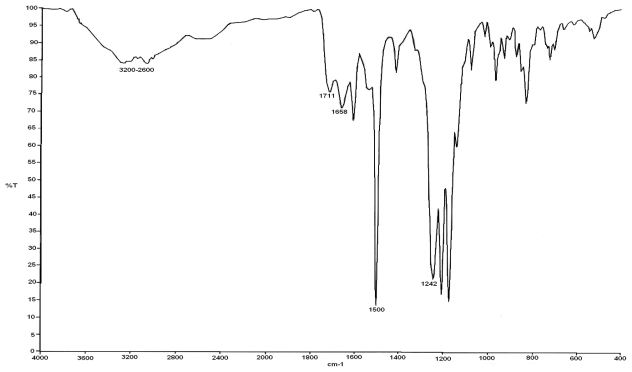
FTIR spectra of the polymer M504. Sample obtained by microwave irradiation at 50 °C and 40 min of irradiation time.

Polymers obtained were characterized by FTIR. Figure 1 presents the FTIR spectra of the polymer M504. According to this result, the formation of the poly(amic acid) structure under our experimental conditions was confirmed by the presence of infrared (IR) absorption bands located at: 3500–2500 cm^−1^ which indicates the existence of -OH group (–COOH), and 1711 cm^−1^ carbonyl group C=O (–COOH stretching vibration), 1658 cm^−1^ carbonyl group C=O (–CONH stretching vibration), 1539 cm^−1^ (N–H bending), 1500 cm^−1^ aromatic ring (C–C stretching), 1242 cm^−1^ indicates the existence of C–N bonds.

### 3.2. Solubility

The solubility of poly(amic acid) sample M504 in dimethylformamide (DMF), dimethyl sulfoxide (DMSO), tetrahydrofuran (THF), acetone, ethanol, hexane, toluene, carbon tetrachloride, and methyl ethyl ketone was investigated at 25 °C following a standard procedure. THF, DMF, ethanol, methyl ethyl ketone and acetone solvents gave good results. Right after the polymer sample came in contact with the liquid, it dissolved completely. Poly(amic acid) sample presents good solubility in DMSO solvent after a period of 30 min. For the remaining solvents tested, the poly(amic acid) did not show any change after 24 h of close contact.

### 3.3. Polymerization

**Figure 2:**
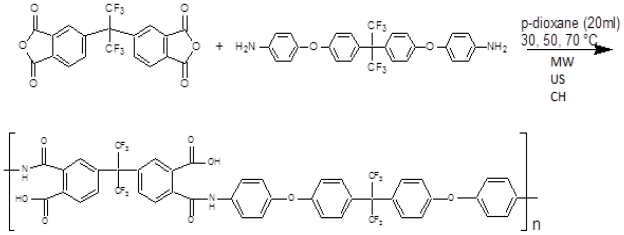
Polymerization reaction scheme.

Polymerization of 4,4′-(hexafluoroisopropylidene)diphthalic anhydride (6FDA) and 4,4′-(hexafluoroisopropylidene)bis(*p*-phenyleneoxy)dianiline (BAPHF) at several reaction times was carried out as depicted, in the chemical reaction scheme in Figure 2. As an example for preparation of sample M302, 1 mmol (0.4442 g) of 6FDA and 1 mmol (0.5184 g) of BAPHF and 20 mL of *p*-dioxane solvent, *T*_bp_ = 102 °C at 760 mmHg, were microwave activated for 20 minutes at 30 °C. Upon reaction time completion, 1 mL of the polymer solution was separated into a 15 mL vial and 10 mL of water were added to fully stop the reaction process. Polymer solid sample was separated from the liquid, washed with water and methanol, and dried at 30 °C for 4 h. The polymer product was weighed to calculate the monomer conversion. The same procedure was repeated for different reaction times and temperatures and with this information, kinetic plots of conversion *versus* reaction time for polycondensation reactions were prepared. Polymerization reactions were carried out in solution to overcome vitrification and gelation which results in reactions becoming diffusion controlled at relative early stage in solid polymerization [34].

**Figure 3:**
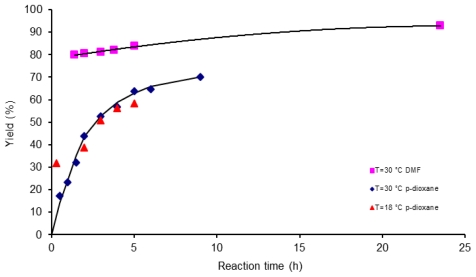
Reaction time and solvent effect on yield (%) for poly (amic acid) 6FDA-BAPHF in *p*-dioxane solvent at two temperatures 18 °C, 30 °C and for poly (amic acid) PMDA-ODA-BTDA in DMF solvent at temperature of 30 °C; reactions activated by conventional heating.

Solvents used in reaction formation of poly(amic acid) play an important role. The most commonly used solvents are dipolar aprotic solvents such as dimethylformamide (DMF), dimethylacetyamide (DMAc), and *n*-methylpirrolidinone (NMP). The strong interaction between the amic acid and the amidic solvent is one of the most important driving forces. Therefore, it is expected that the rate of poly(amic acid) formation is generally faster in more basic and more polar solvents. However, in order to gain a better understanding of the non thermal effects of microwave activation for poly(acid amic) formation, *p*-dioxane solvent, was selected as medium for polycondensation reaction. In Figure 3 values of yield *versus* reaction time for poly(amic acid) 6FDA-BAPHF obtained by conventional heating at 18, 30 °C are compared with experimental data reported by Li *et al*. [35] for poly (amic acid) PMDA-ODA-BTDA synthesized using pyromellitic dianhydride (PMDA), 3,3′,4,4′-benzophenonetetracarboxylic dianhydride (BTDA) and 4,4′-oxydianiline (ODA) in dimethylformamide (DMF) at 30 °C, under conventional heating. As shown in Figure 3, the effect of the polar solvent DMF is apparent, since the yields of the macromolecules are clearly different. Polymer production using a polar solvent DMF is always higher than yield obtained when the same reaction is carried out in *p*-dioxane solvent. After 5 h reaction time, the yield value recorded for system PMDA-ODA-BTDA was 84% in DMF and 63% for system 6FDA-BAPHF in *p*-dioxane solvent.

**Figure 4:**
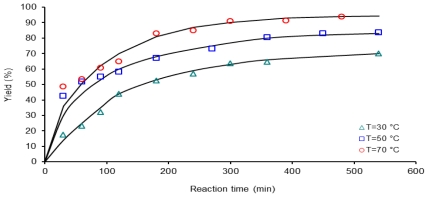
Dependence of yield (%) on the reaction time, 1 mmol of 6FDA-BAPHF, 20 mL of *p*-dioxane solvent at three temperatures 30 °C, 50 °C and 70 °C; reactions activated by conventional heating.

**Figure 5:**
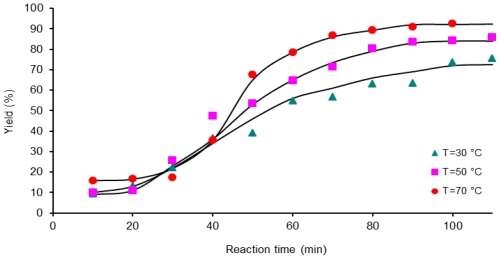
Dependence of yield (%) on the reaction time, 1 mmol of 6FDA-BAPHF, 20 mL of *p*-dioxane solvent at three temperatures 30 °C, 50 °C and 70 °C, reactions activated by microwave heating.

**Figure 6:**
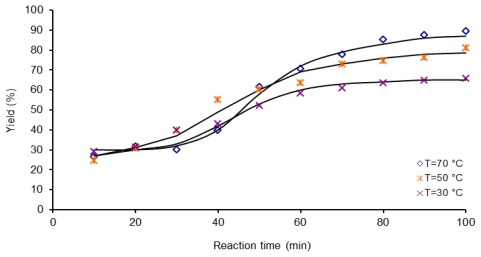
Dependence of yield (%) on the reaction time, 1 mmol of 6FDA-BAPHF, 20 mL of *p*-dioxane solvent at three temperatures 30 °C, 50 °C and 70 °C, reactions activated by ultrasound.

Results for the polymerization reaction for three activation methods, CH, MW and US, at three temperatures, 30, 50, 70 °C are presented in Figures 4–6.

Figure 4 shows the kinetic data of reactions activated by conventional heating at 30, 50 and 70 °C. Polymerization reaction proceeded slowly based upon the first 120 min reaction time where yield does not reach more than 65% monomer conversion at a temperature of 70 °C.

At 30 °C and longer reaction times, more than 300 min, yield increases slightly. It can be explained as a reduction in the probability that two polymeric reactive molecules come together and produce a macromolecule of PAA. For example, it can be observed to augment polymer production from 63% to 70% when the reaction time increases from 300 to 540 min, as in samples C307 and C309.

Kinetic data of three reactions activated by microwave heating at 30, 50 and 70 °C are shown in Figure 5. As expected, in all cases the polymerization proceeded faster than polycondensation activated by conductive heating, yield values increase as irradiation time increases. Thus, a monomer conversion of 55% was obtained at 60 min while 73% yield was reached in 100 min irradiation time at the same temperature of 30 °C; samples M306 and M3010. This trend is also followed by the yield of the reaction when an augment in temperature of polymerization is present. For example, yield goes from 83% at 50 °C to 90.7% at 70 °C in the same reaction time of 90 min; samples M509 and M709. These results are in agreement with values reported by Tang *et al*. [36] for polycondensation of sodium tetrazodiphenyl naphthionate and pyromellitic dianhydride under microwave irradiation, he found that the conversion continued increasing with the lengthening of the irradiation time, the influence of molecular size chains on the reaction activity of the groups, could be neglected when the polymerization degree was not too large. Therefore, the macromolecules condensed continuously, as long as the microwave energy was supplied. In the first 40 min reaction time, the conversion *versus* time behavior for the experiments carried out at 30, 50, 70 °C followed almost the same trend as at longer reaction times, the temperature effect is clear as we can corroborate in Figure 5.

Figure 6 presents kinetic data for three reactions activated by ultrasound. Yields obtained by this activation method are comparable to those obtained by microwave activation experiments. A yield of 87% was reached with 90 min of sonication time, sample U709, if we compare this yield value with the value obtained in the same reaction conditions of temperature (70 °C) and time for sample synthesized by microwave irradiation, sample M709, just a slight difference of 4% was observed. The high yield observed under US conditions can be explained by effective acoustic cavitation, ultrasound comprises sonic waves with frequencies in the range of 0.001 to 107 MHz, when these waves are transmitted through a liquid cavitation takes place [37], it can be defined as the growth and violent collapse of micrometer-sized bubbles. The collapse of these bubbles generates extreme conditions within the core of the bubble, resulting in very high temperatures and pressures, which provide enough energy for polycondensation reaction; very efficient mixing and formation of liquid jets can also occur, due to the rapid motion of fluid [38].

**Figure 7:**
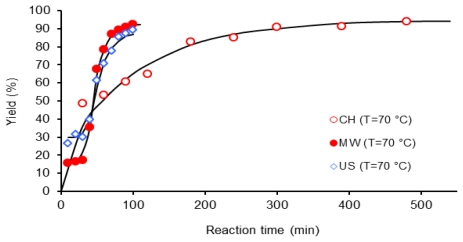
Dependence of yield (%) on the reaction time, 1 mmol of 6FDA-BAPHF, 20 mL of *p*-dioxane solvent at 70 °C, reactions activated by three methods: MO, US, CH.

In Figure 7 values of yield *versus* time for PAA at 70 °C obtained by the three activation methods MW, US and CH are compared. The microwave assisted polycondensation and ultrasound irradiation produced the best polymeric yield, since they reach 85% of monomer conversion in a time of 70 and 80 min respectively (samples M707 and U708) a similar yield was obtained in 240 min when the reaction was activated by conventional heating (CH), sample C706.

With the aim to compare the polycondensation reaction rate constant values for three activation methods, kinetic data were analyzed using a second order kinetic Equation 1 [39].

(1) $$\frac{1}{1-p}=M_{0}kt+1$$

where M_0_ is the monomer concentration at *t* = 0 [mol/L], *p* is the monomer conversion, *k* is a second order rate constant [L/mol s], *t* = time [s].

**Figure 8:**
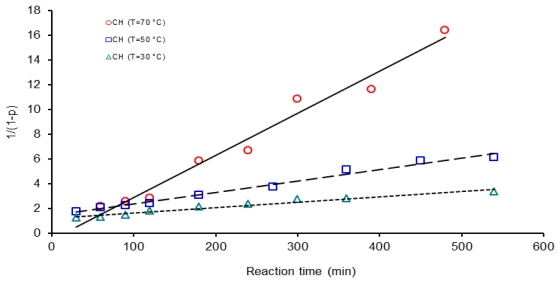
Second order kinetic plot of 1/(1 − *p*) *vs*. reaction time, for 6FDA-BAPHF, 20 mL of *p*-dioxane solvent at three temperatures 30 °C, 50 °C and 70 °C, reactions activated by conventional heating.

**Figure 9:**
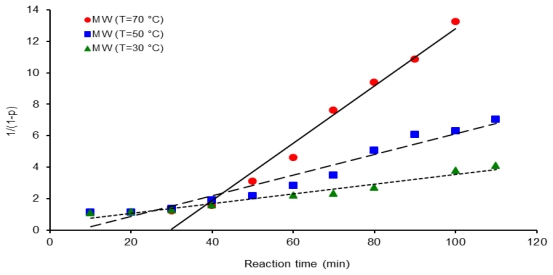
Second order kinetic plot of 1/(1 − *p*) *vs.* reaction time, for 6FDA-BAPHF, 20 mL of *p*-dioxane solvent at three temperatures 30 °C, 50 °C and 70 °C, reactions activated by microwave heating.

**Figure 10:**
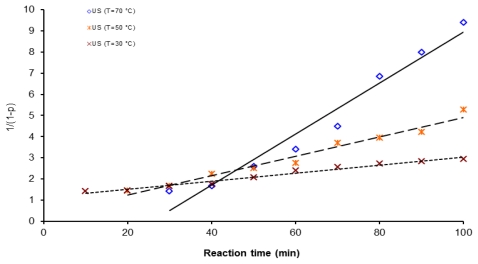
Second order kinetic plot of 1/(1 − *p*) *vs*. reaction time, for 6FDA-BAPHF, 20 mL of *p*-dioxane solvent at three temperatures 30 °C, 50 °C and 70 °C, reactions activated by ultrasound.

Second order kinetic plots of reaction time *vs.* 1/(1 − *p*) for polycondensation reactions at three different temperatures are shown in Figures 8–10. All the kinetic data are fit to this second order kinetic equation. Calculated correlation coefficients values are always higher than 0.9586 and the best values obtained were: 0.9838 for a reaction activated by conventional heating at 50 °C, 0.9791 for microwave assisted polymerization at 70 °C and 0.9758 for the ultrasound assisted experiments at 30 °C.

**Figure 11:**
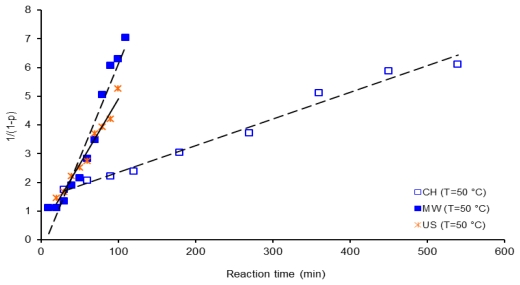
Second order kinetic plot of 1/(1 − *p*) *vs*. reaction time, for 6FDA-BAPHF, 20 mL of *p*-dioxane solvent at 50 °C, reactions activated by the three methods.

Figure 11 illustrates a second order kinetic plot of 1/(1 − *p*) *vs*. reaction time, for 6FDA-BAPHF system at 50 °C, reactions were activated by the three methods, microwaves (MW), samples M501 to M5011, ultrasound (US), samples U501 to U5010 and conventional heating (CH), samples C501 to C5010. As shown in this Figure 11, a rapid acceleration is induced in the synthesis of PAA under US and MW conditions, since slope values for reaction activated by microwaves and ultrasound are higher than slope value obtained when polymerization reaction was activated by conventional heating in all experiments, the reaction conditions were the same.

Table 1 summarized rate constant values *k* [L mol^−1^ s^−1^] for three reaction activation methods, obtained from the slope of the second order kinetic plots, see Figures 8–10. Constant rate values for poly (amic acid) 6FDA-BAPHF synthesized by conventional heating (CH) at 30, 50, 70 °C are of the same order as *k* values reported for several amidation reactions of simple molecules obtained by this activation technique, CH [40], see Table 2. According to the well recognized fact, reaction rate values present a clear temperature dependence, an increment of 20 °C in the reaction conditions almost increases the reaction rate value by a factor of three. For example, rate constant for polymerization reaction activated by ultrasound increases its value from 3 × 10^−3^ to 8 × 10^−3^ [L mol^−1^ s^−1^], when temperature of the system goes from 30 °C to 50 °C. The kinetic results also demonstrated that the rate of the polymerization reaction changes according to the activation method; if we compare the rate constant obtained by conventional heating, 6 × 10^−3^ [L mol^−1^ s^−1^], with a rate constant generated by microwave activation, 30 × 10^−3^ [L mol^−1^ s^−1^], at the same temperature 70 °C, a factor of 5 can be calculated. The reaction rate enhancement under microwave irradiation has been also observed in the emulsion polymerization of styrene early reported by Palacios *et al*. [41], they studied the reaction kinetic on the emulsion polymerization of styrene using microwaves and potassium persulphate as initiator of the reaction, compared the results with data obtained in a traditional conductive experiment, a ratio of *k*_p(MW)_/*k*_p(CH)_ = 26.3 was found. Chia *et al*. [42] reported a reaction rate enhancement of 120% on microwave cure of styrene at 300 watts, under comparable reaction conditions for the thermal cure. On the other hand, Lewis presented experimental evidence that microwave irradiation enhanced imidization reaction of one poly(amic acid) by a factor of 34 at 160 °C compared with the conventional thermal process [34].

According to our experiments the reaction rate constants can be arranged in the following order MW > US > CH.

Nowadays the exact reasons why microwave irradiation enhances chemical processes are still not clear. There is experimental evidence that certain chemical transformations, when carried out at the same measured reaction temperature using microwave or conventional heating, lead to different results in terms of product distribution (selectivity) and yield. These difficult to rationalize effects have been referred to as “specific” or “non-thermal” microwave effects [43].

In order to compare the results obtained by direct microwave heating irradiation with the outcome of a conventionally heated reaction, we have conducted experiments using dioxane as the solvent in our reactions. The macroscopic temperature value have to be taken with reserve, however, since the reaction mixture in our particular case is a low viscosity liquid, magnetic stirring was efficient enough to assure a good distribution of temperature, in this way ensuring that temperature profiles of the system were well controlled. So we are confident that our temperature control is correct.

It is well known that polar substances with high dipolar moment (*μ*, expressed in Debye D) and high dilectric constant (*ɛ*) as dimethylsulfoxide solvent, (*ɛ* = 46.7, *μ* = 3.9 D) or dimethylacetamide solvent (*ɛ* = 37.8, *μ* = 3.7 D) are considered a good media for reactions activated by microwaves, in contrast, *p*-dioxane solvent (*ɛ* = 2.2, *μ* = 0.45 D) can be considered as a low microwave absorbing solvent, since, like other common solvents such as carbon tetrachloride (*ɛ* = 2.24, *μ* = 0 D), benzene (*ɛ* = 2.27, *μ* = 0 D), it has a low dipole moment. Thus, under these conditions most of the microwave energy supplied to the reaction will indeed be absorbed by the polar monomer reagents. We considered experimental values of microwave heating rates for *p*-dioxane being of low significance.

In order to investigate the existence of any non-thermal microwave effect on the condensation polymerization of PAA, Equation 2, was applied. Specific microwave effects have been proposed to be the consequence of wave-material interactions, leading to a decrease in activation energy or change in the pre-exponential factor in the Arrhenius Equation due to orientation effects of polar species maintained in an electromagnetic field.

(2) $$k={Ae}^{-\Delta G_{a}/Rt}$$

*A*: pre-exponential factor; Δ*G*_a_: activation energy [J/mol]; *R*: universal gas constant [J/mol °K].

**Figure 12:**
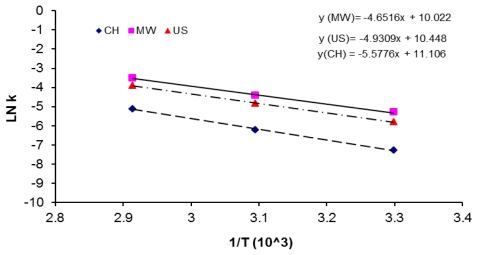
Arrhenius plot second order kinetic data for 6FDA-BAPHF, reactions activated by the three methods.

For comparison purposes activation energies (Δ*G*_a_), reaction rate constants, pre-exponential factors of the Arrhenius equation for the synthesis of PAA by the three activation methods were calculated. The rate constants were plotted, as shown in Figure 12. The calculated activation energies Δ*G*_a_, differences among those calculated values (Δ) and pre-exponential factors (A) for the three activation methods are displayed in Table 1. The pre-exponential factors (A) decrease in the following order 6.6 × 10^4^ (CH) > 3.4 × 10^4^ (US) > 2.2 × 10^4^ (MW) for poy(amic acid) synthesis by three activation methods. This observation is in good agreement with the results reported by Lewis [34] who found that pre-exponential factor A for microwave imidization reaction of poly(amic acid) BTDA-DDS was lower than pre-exponential factor calculated when imidization reaction was carried out by thermal treatment. The values of pre-exponential factors were 4.4 × 10^5^ and 3.95 × 10^10^ for microwave and thermal treatments, respectively.

The microwave and ultrasound activation energy values obtained for the synthesis of PAA are comparable with values obtained under conventional heating for the amidation reaction of simple molecules, 28–34 kJ/mol, reported by Kuznetsov *et al*. [44]. Poly(amic acid) formation is an intermolecular reaction which involves reactive group collisions and reaction, while imide formation is an intramolecular process which is much slower and requires higher activation energy than the former reaction [45], so Δ*G*_a_ values for PAA formation, are lower than Δ*G*_a_ values for imidization reaction of several polyimides, 67–101 kJ/mol, as reported by Yilmaz [46].

From Table 1, it can be seen that Δ*G*_a_ changed in the following order Δ*G*_a_ (MW) < Δ*G*_a_ (US) < Δ*G*_a_ (CH), for polymerization synthesis of PAA. It is necessary to supply 7.7 and 2.3 kJ/mol more energy for polycondensation reaction activated by conventional heating and ultrasound irradiation, than Δ*G*_a_ required for activation by microwave irradiation.

The close comparative analysis of results between conventional heating experiments and microwave assisted polycondensation indicated the presence of a non-thermal microwave effect, according to our analysis, the microwave polycondensation reaction requires a lower activation energy, the polarity is only slightly modified between the ground state (GS) and the transition state (TS) during the course of the reaction and only weak specific microwave effects can be foreseen under this condition. According to Perreux *et al*. [47] specific microwave effects can be expected for the polar mechanism, when the polarity is increased during the reaction from the ground state toward the transition state. The outcome is essentially dependent on the solvent medium and the reaction mechanism. If stabilization of the transition state is more effective than that of ground state, this results in an enhancement of reactivity by a decrease in the activation energy. The transformation of the chemical species in our system is enhancing by a decrease in activation energy Δ*G*_a_. This point is clearly shown by activation energy values calculated and presented in Table 1.

The induction time in a chemical reaction is usually defined as the necessary time to fully initiate the reaction and this step in the process is affected by the heating method. As an example, kinetic line for polycondensation activated by microwave irradiation at 70 °C, Figure 9, crosses the time axis at 30 minutes. Induction times for three methods at three temperatures were observed making this consideration and data values are displayed in Table 1. According to Table 1, induction time for conventional heating activation decreases proportionally as the temperature increases from 30 to 70 °C; however, reaction activated by microwaves and ultrasound do not present this trend. When the system was irradiated with microwaves or ultrasound in a temperature range from 30 to 50 °C induction time decreased as the temperature of the system increases; however, after 50 °C an augment in temperature generates an increment in induction time values. So induction times vary anomalistically with an increment in temperature in the microwave and ultrasound reaction systems. Thus, induction time for polycondensation reaction activated by microwaves at 70 °C was 30 min, which was longer than induction time at 30 °C, 16.6 min, for reaction at the same experimental conditions.

Table 1 also shows the relationship between induction time data and the reaction rate constant for reactions activated by conventional heating, microwave and ultrasound irradiation. It is well known that rate constant for a chemical process activated by conventional heating declines proportionally as the induction time increases; however, this behavior was not observed under microwave and ultrasound activation. Induction time values for experiments under microwave irradiation first decline as constant rate value increases, and then it moves in the opposite direction. On the other hand, when polymerization reaction was activated by ultrasound radiation, constant rate values show a maximum of 8 [L mol^−1^ s^−1^] at 50 °C. It can be see that induction time does not follow a regular path. Similar effects have also been observed for the ring-opening polymerization of ɛ-caprolactone, in toluene solvent, under microwave assisted polymerization at high temperature of 165, 180, 197, 213 and 228 °C [48].

## 4. Conclusions

The synthesis and characterization of poly(amic acid) obtained from 6FDA and BAPHF monomers in *p*-dioxane solvent under conventional heating (CH), microwave (MW) and ultrasound irradiation (US) in a relatively low temperature range were studied to gain a better understanding of the accelerated reaction rates observed under US and MW conditions. It was found that the polycondensation reaction rate decreases in the following order MW > US > CH and that the increased reaction rates observed with US and MW are due to lower activation energies Δ*G*_a_ calculated from the Arrhenius equation. It is suggested that physical effects such as hot spots are important in the accelerated syntheses performed under US and MW conditions. If only this phenomenon is presented in microwave assisted polycondensation of PAA from 6FDA-BAPHF monomers, results must be the same as those obtained by CH activation; however, this is not the case.

Our results indicated that microwave irradiation could activate molecules directly since the microwave energy supplied to the reaction system was absorbed mainly by the monomer reagents. Induction time for conventional heating activation decreases proportionally as the temperature increases. However, for reactions activated by microwaves and ultrasound, the induction times varies anomalistically. Thus, it is reasonable to think that a non-thermal microwave effect is present in our system.

The PAA syntheses performed under US and MW activation may be very promising methods for attaining poly(amic acids) at lower temperatures and in shorter reaction times, which would reduce the energy consumption.

## Figures and Tables

**Table 1:** Kinetic parameters derived from second order kinetics for 6FDA-BAPHF system; rate constant k, activation energy Δ*G*_a_, pre-exponential factor A and induction time *t*_ind_.

Activation Method	T(°C)	*k* × 10^3^ (L mol^−1^ s^−1^)	Δ*G*_a_(KJ/mol)	Δ *	A(L mol^−1^ s^−1^)	Induction time (min)
CH	30	0.7	46.37	7.7	6.6 × 10^4^	250.0
	50	2.0				166.6
	70	6.0				16.6
US	30	3.0	40.99	2.3	3.4 × 10^4^	58.3
	50	8.0				6.66
	70	2.0				21.1
MW	30	5.0	38.67	-	2.2 × 10^4^	16.6
	50	12				3.3
	70	30				30.0

* Δ = Δ*G*_a_ (CH) − Δ*G*_a_ (MW) and Δ*G*_a_ (US) − Δ*G*_a_ (MW).

**Table 2:** Rate constant values for several amidation reactions of simple molecules obtained by conventional heating at 30 °C and −10 °C.

Anhydride	Amine	Solvent	*k* × 10^3^ (L mol^−1^ s^−1^) *
Phthalic anhydride	4-Phenoxyaniline	THF	0.3 a
Phthalic anhydride	4-Phenoxyaniline	Acetonitrile	0.1 a
Phthalic anhydride	Aniline	THF	0.59 b
Tetrahydrophthalic anhydride	4-Phenoxyaniline	Acetonitrile	7.5 a

* Data taken from reference 38;

a equimolar reaction at 30 °C;

b equimolar reaction at −10 °C.
